# Clinical and Social Features of Patients with Eye Injuries Admitted to a Tertiary Hospital: A Five-Year Retrospective Study from Crete, Greece

**DOI:** 10.3390/healthcare11060885

**Published:** 2023-03-18

**Authors:** Elli D. O. Kyriakaki, Efstathios T. Detorakis, Antonios K. Bertsias, Nikolaos G. Tsakalis, Ioannis Karageorgiou, Gregory Chlouverakis, Emmanouil K. Symvoulakis

**Affiliations:** 1 Clinic of Social and Family Medicine, School of Medicine, University of Crete, 71003 Heraklion, Greece; 2 Department of Ophthalmology, University Hospital of Heraklion, Crete, 71500 Heraklion, Greece; 3 Clinic of Social and Family Medicine, Faculty of Medicine, University of Crete, 71003 Heraklion, Greece; 4 Department of Ophthalmology, General Hospital of Ierapetra, 72200 Ierapetra, Greece; 5 School of Medicine, University of Crete, 71003 Heraklion, Greece; 6 Department of Social Medicine, Biostatistics Lab, School of Medicine, University of Crete, 71003 Heraklion, Greece

**Keywords:** eye injury, occupation, retrospective, visual outcomes, prevention

## Abstract

Eye injuries are a major cause of visual disability worldwide and may present a burden to both quality of life of the sufferers and healthcare services. The aim of this study was to extract and triangulate information on the demographic, clinical, and social features of eye-injured adult patients admitted to a tertiary hospital in Greece. The design was a five-year retrospective study of eye-injured adult patients, admitted to the General University Hospital of Heraklion, Crete (GUHH), the single tertiary referral hospital on the island. Drawing the profile of eye-injured patients may add to future health planning. Data collected from 1 January 2015 to 31 December 2019, such as sociodemographic features and clinical information, were extracted. One hundred twenty-eight patients were included. Of those, there was no available information on activity during injury for 6 patients, 78 (60.9%) had work-related ocular injuries, and 44 (34.4%) had non-work-related ocular injuries. Patients with no current formal employment, those who were retired, and formally unemployed and manual force workers had the higher rates of work-related injuries. The most common work-related injuries were closed globe injuries, specifically contusions, while ruptures and penetrating wounds were the most frequent of the open globe injuries. Within the univariate analyses, work-related eye injuries were significantly associated with male gender, middle age, and the place related to daily work activity. Determinants of poor final visual acuity (VA) were the initial VA, the type of injury (*p* < 0.0001), the distance of the place of residence from the hospital, and the time to hospital admission (*p* < 0.013). In a multivariate analysis, referred patients and those with open globe injuries arrived at hospital after a two-hour interval compared with those who were not referred and those with closed globe injuries (*p* ≤ 0.05). A reduction in the time to hospital admission deserves further attention. The interconnection of community and health system services through a capacity increase and networking needs further research in order to obtain targeted and viable access for eye-injured patients.

## 1. Introduction

Ophthalmic traumas include injuries of the eyelids, corneal and conjunctival abrasion, contusion of the globe, rupture, intraocular hemorrhage, optic nerve and orbital trauma, and retinal or chorio-retinal trauma [1]. According to Birmingham Eye Trauma Terminology (BETT), ophthalmic trauma can be classified into Open and Closed globe injuries [2]. Open globe injuries are full thickness wounds that are grouped into ruptures and lacerations, while lacerations are further classified as penetrating and perforating wounds and intraocular foreign bodies [2]. Closed globe injuries are sub-grouped into lamellar lacerations and contusions of the eye globe [2]. Eye injuries are a major cause of avoidable visual disability worldwide. They can be caused by falls, car accidents, chemical burns, or assaults, or during agricultural, sport, or occupational activities, among other methods [3]. They represent a significant burden to healthcare systems [4] and can reduce the quality of life of sufferers [5]. Of the 55 million estimated ocular injuries occurring yearly, approximately 750,000 require hospital admission, with 250,000 being open-globe injuries [1].

Ophthalmic trauma is associated with various factors, such as the geographic location, culture, and socioeconomic status of the population [1,6]. Many studies show that most eye wound injuries are work-related [7,8] and mainly occur among men as they are usually exposed to more higher-risk activities than women are [9]. A 15-year retrospective study from Portugal revealed that, apart from gender and the location of the injury, the economic situation may be included as a high-risk determinant [10]. The study showed that ocular eye injury incidents were related to Portugal’s economic recession period, and the authors stated that job uncertainty and work-related pressure may be associated with this rise [10]. Additionally, the same study showed that 7.3% of the ocular injuries were related to substance or alcohol use [10]. Both elements link social and economic determinants with eye injuries.

Moreover, ocular injuries have psychological and economic impacts on individuals, because they lead to loss of work capacity and productivity limitations [11]. They also present an economic burden to the insurance system of a country, because of the constantly increasing hospitalization costs [12,13]. General practitioners and occupational health providers may be able to play a role in the prevention of these injuries and limit complications by providing a care network for mild eye injuries and by giving prompt referrals for severe cases [14]. Regarding the prevention of occupational eye injuries, employers and occupational health and safety professionals should develop personalized messages for the proper use of protective eye equipment and other measures in workplaces.

This study aims to extract and triangulate information regarding the demographic, clinical, and social features of eye-injured adult patients admitted to the General University Hospital of Heraklion (GUHH), Crete, Greece. Drawing the profiles of eye-injured patients can offer some baseline input for future service adjustments.

## 2. Materials and Methods

### 2.1. Study Design, Population, and Ethics Approval

Crete is relatively isolated from mainland Greece and has a fully supported healthcare system with primary healthcare centers and secondary and tertiary hospital units. The single tertiary hospital in the geographical region of Crete provides medical services to a population of over 632,674 permanent inhabitants living in all four prefectures of Crete in a catchment area of 8336 km^2^. In the summer the population grows significantly due to tourism. We conducted a retrospective study of patients admitted to the GUHH, with ocular injuries from 1 January 2015 to 31 December 2019, prior to the COVID-19 pandemic. Therefore, COVID-19 did not affect the hospitalization length, doctor availability, or overall capacity of the hospital. The medical records of patients who presented to the Ophthalmology emergency department were screened and reviewed by the first author to determine their eligibility. Participants included in the study were adults with ocular injuries severe enough to require hospitalization at the ophthalmology department. The total sample included 128 patients. The gender, age, occupation, family status, nationality, and insurance status of the patients as well as several clinical variables were analyzed. Missing information was added, when possible, via telephone interviews.

The GUHH is the only reference center on Crete and is on duty every second day. All departments are active to deal with emergencies, thus, there is no process or management variation between weekdays and weekends. Most participants included in the study were insured. However, in Greece, all patients, whether they are insured, uninsured, or foreigners/tourists, benefit equally from emergency care. Due to the available on-call system, most emergency cases that need surgical treatment undergo interventions without a prolonged delay. For instance, in the case of vitreoretinal surgery, patients are hospitalized for posturing, and the surgeon is called to perform the surgery in due time. All procedures associated with serious eye injuries are performed under general anesthesia.

Age was classified into three categories: 18–40, 41–66, and over 67 years. Work activity was defined as any paid formal professional activity with a job contract agreement with an employer. Secondary occupational activities were defined as any working activities without a job contract but conducted for indirect profit. These were mainly related to agriculture, which is a socio-cultural commonality for the inhabitants of Crete, even after retirement.

The source of injury was coded into four categories: solid object injury, chemical burns, livestock–agricultural activity, fall and sport leading injuries. The classification of open and closed globe injuries was based on the BETT criteria [2] and the Ocular Trauma Classification Group [15,16]. The Initial and Final Visual Acuity (VA) were used to define the visual outcomes. Additionally, a conversion of all VAs from the Snellen chart to the Logarithm of the Minimum Angle of Resolution (LogMAR) was applied. The geometric mean of the LogMAR values was used for the statistical analysis and comparisons of different datasets and variables [17]. Moreover, the LogMAR measurement was used for research purposes, because it has the potential to accurately calibrate low VA and to practically facilitate its analysis [18,19]. VA was assessed on presentation at the clinic and upon recovery. The final VA was the last value recorded in a patient’s record file following hospitalization in the ophthalmology department or after outpatient follow-up. Eleven patients with initial and final VA scores of 10/10 on the Snellen scale (LogMAR = 0.00) were not included. VA was arbitrarily coded into poor (≤0.5/10 or ≤20/400 on the Snellen scale, ≥1.3 on the LogMAR scale) and not poor (>0.5/10 or >20/400 on the Snellen scale, <1.3 in LogMAR scale) [20]. Time to admission was coded as ≤2 or >2 h. The residence distance from the hospital (in kilometers) was categorized as 0–20 km, 21–60 km, or over 61 km. Given the variance in the road network and island geography, a linear relation between time and distance could not be estimated.

The patients’ insurance statuses were coded into four main categories: public, social, agricultural, and self-employment insurance, according to the Greek insurance system, whilst data were also recorded for uninsured patients. Data on the cost of care included the cost of all days for hospitalized patients. These were provided by the pertinent administrative authorities. The cost was classified into categories of less than EUR 500; EUR 501–1000; EUR 1001–1500; and over EUR 1501.

Children and adolescents aged under 18 years were not included. Patient data were collected according to the guidelines of the Declaration of Helsinki, assuring confidentiality, and the study was approved by the Scientific Council of the 7th Health District of Crete (Prot. Number:17/30-10-2019) and the Scientific Ethics and Deontology Committee of the University of Crete (Prot. Number:28/07-02-2020).

### 2.2. Statistical Analysis

Descriptive statistics were used to summarize all variables. An analysis between work-related injuries and selected variables was performed using Pearson’s chi-square test. A comparison between the initial and final LogMAR performance (poor vs. not poor) was performed using the McNemar test. An analysis of the initial LogMAR values, the delay time, and the risk category was performed using the nonparametric Mann–Whitney test. An analysis of the number of days of hospitalization (0–7 days vs. 8+ days) and certain parameters was performed using Pearson’s chi-square test and a simple logistic regression. An analysis of the delay time (≤2 h vs. >2 h) and selected factors was performed using Pearson’s Chi-Square test. Finally, an analysis of the final LogMAR (poor vs. not poor) and selected variables was performed using Pearson’s Chi-square test and a simple logistic regression. All continuous variables were checked for normality using both histograms and the Kolmogorov–Smirnov Test. A multiple logistic regression model was performed with the dependent variable being the time of admittance (≤2 h vs. >2 h). Independent variables used in the model were the initial visual acuity (not poor vs. poor), the final visual acuity (not poor vs. poor), the type of injury (open globe injury vs. closed globe injury), and referral to hospital by any doctor (yes/no). The adopted level of significance was *p* = 0.05, and data were analyzed with the statistical software IBM SPSS, version 24.3.

## 3. Results

Among the 128 ocular injury patients, 113 (88.3%) were males and 15 (11.7%) were females. The mean age was 52.39 ± 17.64 years. The majority of patients did not have an active employment status (39.4%), but most of them (30.7%) had retired and were involved in secondary non-formal agricultural work, while 8.7% were formally unemployed. Fifty percent of patients had social insurance/self-employment insurance (Table 1). The BETT system classifications [2] of the ocular injuries reported in our study analysis are shown in Table 2.

Univariate comparisons were conducted between work-related/non-work-related injuries and several variables. Most injuries (78, 60.9%) were work-related, while 44 (34.4%) were non-work-related, and 6 patients (4.7%) had injuries with an unknown reason and were thus removed from this analysis. A higher proportion of men (98.7%) than women (1.3%) had work-related eye trauma (*p* < 0.0001) (Table 3). Statistically significant differences in work-related eye injuries among different age groups were observed, with a higher proportion of injuries occurring in those aged 41–66 years (56.4%), compared with those aged 18–40 years and those older than 67 years (*p* = 0.014). The higher rates of work-related eye injuries, approximately 30%, were observed among manual force workers and in those with no currently formal employment. Significant differences between work- and non-work-related eye injuries in terms of the source and the place of injury were also observed (*p* < 0.0001). Solid-object injuries, livestock and agricultural injuries, and chemical burns were more common in the work-related injury subgroup (48.1%, 31.2%, and 18.2%, respectively). Solid-object injuries were more common in the non-work-related group (38.6%), followed by falls and sports injuries (34.1%). As for the place of injury, work-related ocular injuries occurred during formal or non-formal work activities, as previously defined, whereas most non-work-related injuries occurred at home (*p* < 0.0001, Table 3). Most patients with work-related eye injuries did not wear protective eye devices (PED) while performing their duties (*n* = 57, 90.5%).

Within the current patient study group, there were registered 31 lesions of vitreous hemorrhage, 30 lesions of traumatic cataract, 22 lesions of partial iris loss, and one lesion of total iris loss. Seven lesions of retinal detachment were recorded. No case of endopthalmitis was reported.

There was no significant association of the duration of hospitalization with gender, age, or occupation. On the contrary, patients with closed globe injuries had increased rates of hospitalization over 8 days (33, 73.3%) compared with those with open globe injury (12, 26.7%) (*p* < 0.0001). Moreover, the season was associated with the duration of hospitalization. In terms of the hospitalization length, eye injuries that occurred in autumn differed when compared to those occurring in spring (<8 days of hospitalization: 17.8% in autumn vs. 37.8% in spring); (*p* = 0.041). The hospitalization length did not show a statistically significant correlation with the distance from the hospital (Table 4).

Univariate comparisons between the time to hospital admission and specific variables are presented in Table 5. There was a statistically significant correlation between the initial and final VA and the time to admission. Patients who arrived at the hospital after more than two hours were more frequently assessed as having “poor” initial and final VA than those admitted within two hours (Table 5). Moreover, patients admitted after two hours had a higher likelihood of undergoing surgical intervention compared to those admitted in less than two hours (63.4% vs. 24.1%, *p* < 0.0001). There were significantly more patients with a poor final VA following delayed admission compared with those admitted in less than two hours (23.9% vs. 7.3%, *p* = 0.013). In addition, the cost of hospitalization was increased for patients who took over two hours to be admitted (*p* = 0.010), and the same was true for the median personal expenses (*p* = 0.010). Patients referred from private practitioners or public units were admitted significantly later than those who were not referred (*p* < 0.0001, Table 5).

Comparisons between the final VA (not poor/poor) and selected factors are presented in Table 6. The final VA was significantly associated with the initial VA. Patients who did not have a poor initial VA also did not have a poor final VA (74.8%, *p* < 0.0001). Closed globe injuries had more “not poor” final visual outcomes compared with open globe injuries (65.4% vs. 34.6%; *p* < 0.0001). Additionally, patients who lived at a distance of greater than 61 km away from the hospital, and were thus likely admitted late, had a higher percentage of poor outcomes compared with those who lived closer (65.0%, *p* = 0.005). Participants with no currently formal employment had a greater rate of poor final visual outcomes (71.4%) compared with the rest of the occupational categories (manual force workers, farmers/locksmiths and private/public sector employees, and self-employed) (*p* = 0.031).

The results from a multiple logistic regression analysis indicated that the initial and final visual acuity were not significant predictors of the time to hospital admittance (≤2 h vs. >2 h; *p* > 0.05 for both). On the other hand, patients with an open globe injury had 2.78 times higher odds of hospital admittance in >2 h compared to patients with a closed globe injury (OR 2.718; 95% CI from 1.001 to 7.740; *p* = 0.050). Finally, patients who were referred to the hospital from other health-care settings had 23.9 times higher odds of hospital admittance in >2 h compared to patients without a referral (OR 23.94; 95% CI from 6.534 to 87.76; *p* < 0.0001).

## 4. Discussion

This study provides the first analysis of work-related ophthalmic trauma on the Mediterranean island of Crete. Closed globe injuries were the most common type of work-related ocular injury, whilst 4 out of 10 were open globe cases. This is similar to the results reported by another Greek study in 2005 [21]. Closed globe injuries were also associated with increased rates of hospitalization for ≥8 days compared with open globe injuries (73.3% vs. 26.7%, *p* < 0.0001). This finding is in contrast with that of another study, where the most common duration of hospital stay was 3 to 5 days for all types of globe injury [22]. Because closed injuries are managed conservatively, the hospitalization duration may be prolonged. Additionally, as many patients were older than 50 years of age, their health status or overall pharmacotherapy profile might have influenced the duration of hospital stay.

A final not poor VA was found to be strongly correlated with a not poor initial VA, as shown in other studies [23,24]. Comparable results have emerged from a prospective data collection branch study that has been recently published [25]. Open globe injuries were more frequently related to a poor final VA compared to closed globe injuries, showing that visual outcomes depend on the clinical characteristics and the nature of eye damage [23,24]. Within the present study, patients with vitreous hemorrhage received a two-month eye drop medication prescription. Follow-up monitoring, to assess vitreous hemorrhage absorption, was scheduled for these patients. Those with persistent vitreous hemorrhage, after the two-month period, underwent vitrectomy. Patients with traumatic cataract underwent lensectomy. Patients with iris loss underwent iridoplasty. Finally, patients with retinal detachment underwent surgical restoration.

Most of the eye injuries were work-related (60.9%), as observed by other authors [21,26,27]. Manual force workers (construction, welding, electric work, plumbing, farming) formed one of the most commonly affected groups among the admitted patients (29.5%), indicating that these patients have a higher risk of sustaining an eye injury, as highlighted by other studies [21,28,29,30]. A large proportion of patients were retired and spent their spare time in agricultural labor, which also involves high-risk activities [29,30]. Occupation was also strongly correlated with poor final VA, as shown in another study [24].

Most sufferers with work-related injuries had not used protective measures (90.5%), as reported in other studies [21,27]. It is therefore possible that a significant number of eye injuries could be prevented or avoided. However, any effort geared towards increasing PED use compliance should take into consideration the specific social and occupational features of the residents of that region [31], as the occupational characteristics of each region’s population are very important in determining the major sources of ocular injury in that area [1,9,30].

The mean age of the patients was 52.39 ± 17.64 years, which is older than what has been reported in other studies [21,27]. This could be attributed to the fact that, in Crete, many people own properties with crops and work there unofficially after retirement. Injuries that occurred during spring and summer were associated with increased rates of hospitalization over 8 days. In rural Crete, many agricultural activities are carried out during those seasons, thus, more serious eye injuries tend to occur then.

In the univariate analysis, patients admitted to the hospital more than two hours after the occurrence of the injury had poorer initial and final VA compared to those admitted in less than two hours (*p* = 0.010 and *p* = 0.013, respectively). Thus, the final VA depends, among the other factors highlighted in our study, on early hospital admission. This is in line with the results of other studies [23,32]. The multivariate analysis did not confirm this finding. The use of larger sample size groups and different time cut-offs may offer answers in future research efforts. A study conducted in Bosnia did not find an association between the final VA and the time to hospital admission [33]. A recently published study showed that a delay of almost 4 h caused by interhospital transfer led to a lower final VA [34].

Patients with a delayed admission had increased rates of surgical interventions and an increased cost of care and personal cost. To the authors’ current knowledge, few studies have investigated the relationship between time to admission and economic parameters. As shown in Table 5, both the overall cost of care and the out-of-pocket payment of patients were significantly lower when the time to admission was less than 2 h (*p* = 0.010). It could, therefore, be assumed that late eye injury management is likely to lead to poorer outcomes [32], and this may be reflected in the cost of care and personal expenses for the sufferers and the healthcare system. A study from the United States found that the cost of care was higher for patients from high-income households, probably, as discussed by the authors, because health providers were more willing to order tests and because the patients themselves demanded a higher care level [12].

The multiple regression analysis showed that patients with open globe injuries had significantly higher odds of being admitted to the hospital in >2 h from the incident compared to those with closed globe injuries, while another study showed that the median time to hospital admittance was 4 h for patients with an open globe injury [35]. A possible explanation for this is that open globe injuries are likely to occur in the context of more complex trauma, which may lead to a process involving service prioritization, and this may influence the time to arrival at the hospital. The GUHH is on duty every second day, so we cannot exclude the fact that visit timing may have been influenced by this parameter in some cases. For closed globe eye care, access may directly emerge, and time of arrival may be anticipated. It also appears that referred patients are more likely to arrive at the hospital more than two hours after the injury than those who are not referred. Both findings show that the initial assessment and management of ocular injuries deserve an evidence-based design in terms of the roles, flows, and care network, as this might increase the viability of moving patients from community and primary settings to tertiary care.

### 4.1. Implications

Primary care physicians can assist with early hospital admission by assessing and promptly referring patients with severe ocular injuries to the hospital while managing mild cases. This can lead to earlier recognition, and therefore better visual outcomes of serious ocular injuries as well as appropriate management of mild ocular injuries without burdening large referral centers.

PED use compliance was minimal in our study. To increase compliance, the special geographical, occupational, and cultural characteristics of a region should be taken into consideration when designing prevention and protective measures [36]. Occupational health and safety professionals could contribute to the prevention of ocular injuries by educating and counseling workers regarding the importance of using PED in the workplace. A focus on rural areas should be given to inform low-educated, elderly, and residents about the risk of not wearing PED, while local municipalities could provide suitable and well-fitted protective equipment, as a public health measure initiative.

### 4.2. Limitations

This was a retrospective observational study conducted in Crete; thus, the study results are not representative of the whole country. The study included hospitalized patients, therefore, patients with less severe injuries that were managed in local first aid health units or secondary hospitals were not recorded. Considering that the study’s data reflect a pre-COVID-19 period, there were no irregularities regarding the hospitalization length, doctor availability, or overall capacity of the hospital. Additionally, up to the study’s end date, COVID-19 did not affect access or greatly influence possible follow-up consultation, as mentioned in other studies [37,38]. However, it was difficult to collect data retrospectively due to a ban on physical hospital access and the strict measures undertaken for COVID-19, because the data record search and collection were performed during the pandemic. Recall biases cannot be excluded for missing information that was retrieved via phone calls.

## 5. Conclusions

This study concluded that most cases of ocular injury requiring hospitalization in Crete were work-related. Those with no current formal employment, those who were retired or formally unemployed, and manual force workers had higher rates of work-related injuries. Patients with open globe injuries were more likely to attend hospital in >2 h compared to patients with closed globe injuries. Finally, patients who were referred from other health-care settings had an increased likelihood of accessing the hospital in >2 h compared to patients without a referral. Major efforts to improve the initial management of ocular injuries should include an evidence-based design of the roles, flows, and care network to increase the interconnectivity of services.

## Figures and Tables

**Table 1:** Socio-demographic characteristics of eye injuries (N = 128).

		*n* * (%)
Gender		
	Male	113 (88.3%)
	Female	15 (11.7%)
Age group		
	18–40	37 (28.9%)
	41–66	59 (46.1%)
	66+	32 (25.0%)
Family status		
	Married	107 (88.4%)
	Unmarried, divorced, widow	14 (11.6%)
Nationality		
	Greeks	101 (79%)
	Foreigners	27 (21%)
Occupation		
	Manual force workers	28 (22%)
	Farmers/livestock workers	18 (14.2%)
	Self-employed/private-public sector employees	31 (24.4%)
	With no currently formal employment	50 (39.4%)
Insurance status		
	Public insurance	19 (14.8%)
	Private insurance	3 (2.3%)
	Agricultural insurance	34 (26.6%)
	Social insurance/self-employment insurance	64 (50%)
	Uninsured	8 (6.3%)

* Total counts may differ due to missing values.

**Table 2:** Work-related and non-work-related ocular trauma cases stratified from our data collection, according to the Birmingham Eye Trauma Terminology (BETT) [2].

	Work-Related	Non-Work-Related
	*n* *	*n* *
PATIENTS WITH OCULAR INJURIES	78	44
* PATIENTS WITH CLOSED GLOBE INJURIES *	48	23
* Chemical burn lesions *	14	6
* Contusion lesions *	22	12
* Partial thickness wound lesions *	15	7
* PATIENTS WITH OPEN GLOBE INJURIES *	30	21
* Rupture lesions *	29	19
* Full thickness wound lesions *		
* Perforating wound lesions *	28	19
* Penetrating wound lesions *	29	19
* Intraocular foreign body lesions *	7	5

* Number of lesions may differ from the number of patients because they may be more than one lesion per patient.

**Table 3:** Demographic and injury characteristics of work- and non-work-related eye injuries (N = 122 *).

		Work-Related	Non-Work-Related	*p*-Value
		N ** (%)	N ** (%)	
Gender				<0.0001
	Male	77 (98.7%)	31 (70.5%)	
	Female	1 (1.3%)	13 (29.5%)	
Age groups				0.014
	18–40	19 (24.4%)	15 (34.1%)	
	41–66	44 (56.4%)	13 (29.5%)	
	67+	15 (19.2%)	16 (36.4%)	
Occupation				0.016
	Manual force workers	23 (29.5%)	4 (9.1%)	
	Farmers/livestock workers	12 (15.4%)	6 (13.6%)	
	Self-employed/private-public sector employees	19 (24.4%)	9 (20.5%)	
	With no currently formal employment	24 (30.8%)	25 (56.8%)	
Days of hospitalization		5 (1, 26; 6)	6 (1, 22; 7)	0.323
Surgery				0.448
	Yes	33 (42.9%)	22 (50.0%)	
	No	44 (57.1%)	22 (50.0%)	
Cost of care		701 (60, 2615; 551)	799 (188, 2203; 548)	
Type of injury				0.319
	Closed globe injuries	48 (61.5%)	23 (52.3%)	
	Open globe injuries	30 (38.5%)	21 (47.7%)	
Source of injury				<0.0001
	Solid object injury	37 (48.1%)	17 (38.6%)	
	Chemical burns	14 (18.2%)	8 (18.2%)	
	Livestock and agricultural injuries	24 (31.2%)	4 (9.1%)	
	Falls and sport injuries	2 (2.6%)	15 (34.1%)	
Place of injury				<0.0001
	Daily work activity (formal or non-formal)	69 (89.6%)	0 (0.0%)	
	Road and traffic accidents	0 (0.0%)	5 (11.9%)	
	Assaults	0 (0.0%)	1 (2.4%)	
	Sport activities	0 (0.0%)	2 (4.8%)	
	Domestic and other	8 (10.4%)	34 (81.0%)	
Use of protective eye devices				0.823
	Yes	6 (9.5%)	2 (8.0%)	
	No	57 (90.5%)	23 (92.0%)	

* 6 patients were excluded due to having a non-specified reported activity during injury. ** Total counts may differ due to missing values.

**Table 4:** Variables associated with hospitalization (N = 128).

	Hospital Stay (0–7 Days)	Hospital Stay (8+ Days)	*p*-Value
	*n* * (%)	*n* * (%)	
Gender			0.117
Male	76 (91.6%)	37 (82.2%)	
Female	7 (8.4%)	8 (17.8%)	
Age groups			0.268
18–40	26 (31.3%)	11 (24.4%)	
41–66	40 (48.2%)	19 (42.2%)	0.799
67+	17 (20.5%)	15 (33.3%)	0.145
Occupation			0.189
Manual force workers	21 (25.6%)	7 (15.6%)	
Farmers/livestock workers	23 (28.0%)	8 (17.8%)	0.943
Self-employed/private-public sector employees	10 (12.2%)	8 (17.8%)	0.174
With no currently formal employment	28 (34.1%)	22 (48.9%)	0.100
Type of injury			<0.0001
Closed globe	21 (25.3%)	33 (73.3%)	
Open globe	62 (74.7%)	12 (26.7%)	
Season			0.041
Spring	14 (16.9%)	17 (37.8%)	
Summer	20 (24.1%)	11 (24.4%)	0.128
Autumn	28 (33.7%)	8 (17.8%)	0.007
Winter	21 (25.3%)	9 (20.0%)	0.053
Time of admittance			0.082
≤2 h	40 (49.4%)	15 (33.3%)	
>2 h	41 (50.6%)	30 (66.7%)	
Residence distance from hospital (km)			0.144
0–20	34 (41.5%)	14 (32.6%)	
21–60	26 (31.7%)	10 (23.3%)	0.889
61+	22 (26.8%)	19 (44.2%)	0.097

* Total counts may differ due to missing values.

**Table 5:** Injury and cost outcomes related to the time of access (N = 126 *).

		Time of Admittance to Hospital (≤2 h)	Time of Admittance to Hospital (>2 h)	*p*-Value
		N ** (%)	N ** (%)	
Initial visual acuity (LogMAR scale)				0.010
	Not poor	41 (74.5%)	37 (52.1%)	
	Poor	14 (25.5%)	34 (47.9%)	
Final visual acuity (LogMAR scale)				0.013
	Not poor	51 (92.7%)	54 (76.1%)	
	Poor	4 (7.3%)	17 (23.9%)	
Days of hospitalization				0.082
	0–7	40 (72.7%)	41 (57.7%)	
	8+	15 (27.3%)	30 (42.3%)	
Surgical intervention				<0.0001
	Yes	13 (24.1%)	45 (63.4%)	
	No	41 (75.9%)	26 (36.6%)	
Cost of care				0.010
	≤500 €	31 (57.4%)	20 (28.2%)	
	501–1000 €	17 (31.5%)	33 (46.5%)	
	1001–1500 €	3 (5.6%)	9 (12.7%)	
	1501+	3 (5.6%)	9 (12.7%)	
Pocket payments				
	Median(min, max; ΙQΡ)	80 (0, 10.000; 150)	150 (0, 15.000; 600)	0.010
Referral by				<0.0001
	Private practice	0 (0.0%)	16 (23.2%)	
	Public practice	3 (5.5%)	28 (40.6%)	
	None	52 (94.5%)	25 (36.2%)	

* 2 patients were excluded due to having a non-specified time of admittance. ** Total counts may differ due to missing values.

**Table 6:** Factors associated with visual outcomes (N = 128).

	Final Visual Acuity (LogMAR Scale)	Final Visual Acuity (LogMAR Scale)	*p*-Value
	Not Poor	Poor	
	*n* * (%)	*n* * (%)	
Initial visual acuity (LogMAR scale)			<0.0001
Not poor	80 (74.8%)	0 (0.0%)	
Poor	27 (25.2%)	21 (100.0%)	
Type of injury			<0.0001
Closed globe injury	70 (65.4%)	4 (19.0%)	
Opened globe injury	37 (34.6%)	17 (81.0%)	
Occupation			0.022
Manual force workers	26 (24.5%)	2 (9.5%)	
Farmers/livestock workers	16 (15.1%)	2 (9.5%)	0.644
Self-employed/private-public sector employees	29 (27.4%)	2 (9.5%)	0.916
With no currently formal employment	35 (33.0%)	15 (71.4%)	0.031
Residence distance from hospital (km)			0.003
0–20	45 (42.9%)	3 (15.0%)	
21–60	32 (30.5%)	4 (20.0%)	0.431
61+	28 (26.7%)	13 (65.0%)	0.005
Time of admittance to hospital			0.013
≤2 h	51 (48.6%)	4 (19.0%)	
>2 h	54 (51.4%)	17 (81.0%)	
Surgical intervention			<0.0001
No	68 (64.2%)	1 (4.8%)	
Yes	38 (35.8%)	20 (95.2%)	

* Total counts may differ due to missing values.

## Data Availability

The data that support the findings of this study are available on request from the corresponding author. The data are not publicly available due to privacy or ethical restrictions.
